# Supplementary material for: Redox-switchable breathing behavior in tetrathiafulvalene-based metal–organic frameworks
Source: Nat Commun. 2017 Dec 8;8:2008. doi: 10.1038/s41467-017-02256-y (PMC5722820; doi:10.1038/s41467-017-02256-y)
Supplement: Supplementary file 1 — Supplementary Information [file 41467_2017_2256_MOESM1_ESM.pdf]

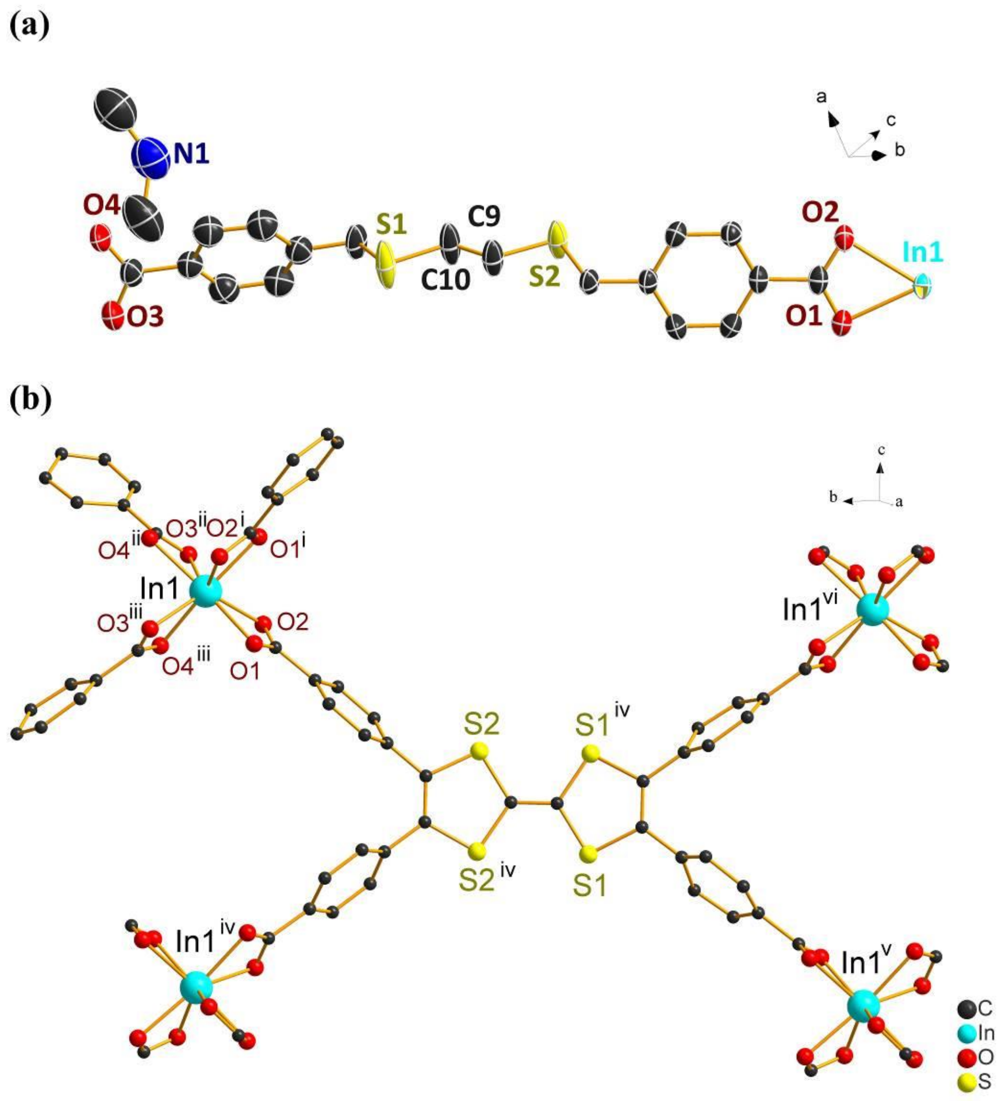


**Supplementary Figure 1.** (a) The asymmetric unit of compound **1**. Displacement ellipsoids are drawn at the 50% probability level. (b) The coordination environment of TTFTB$^{4-}$ and In$^{3+}$ ions in compound **1**. H atoms are omitted for clarity. Symmetry transformations used to generate equivalent atoms: i (-x, y, 1.5-z), ii (0.5-x, 0.5+y, 0.5+z), iii (-0.5+x, 0.5+y, 1-z), iv (x, y, 1-z), v (0.5+x, -0.5+y,1- z), vi (0.5+x, -0.5+y, z).

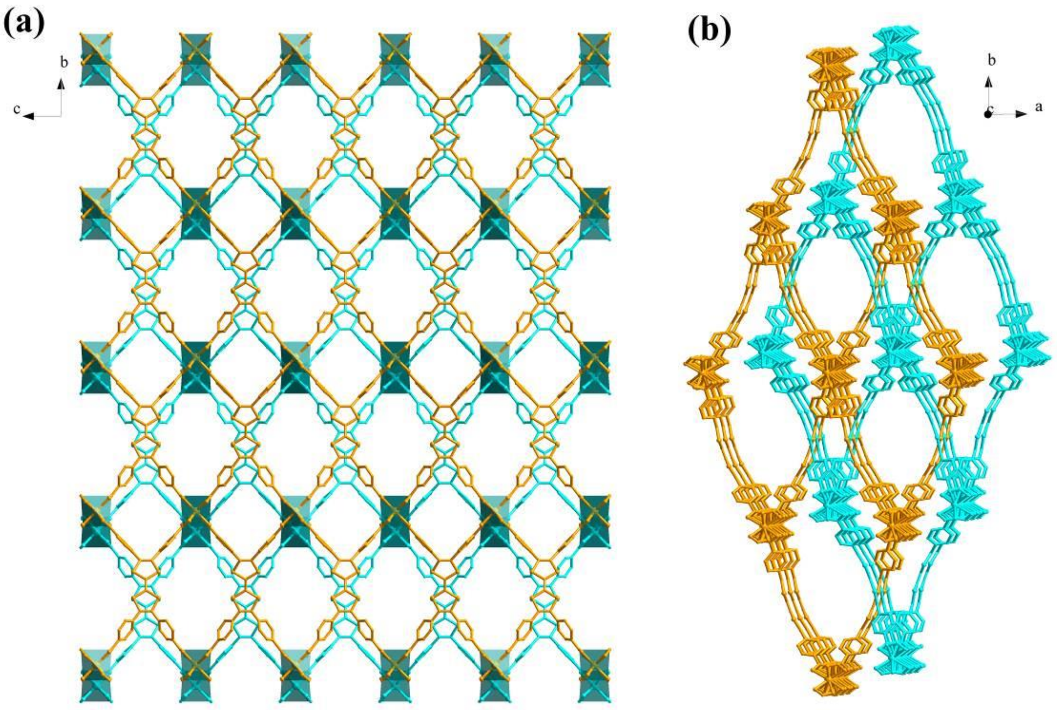


**Supplementary Figure 2.** The 2-fold interpenetrating 3-D framework of compound **1** view in the ***a*** (a) and ***c*** (b) direction.

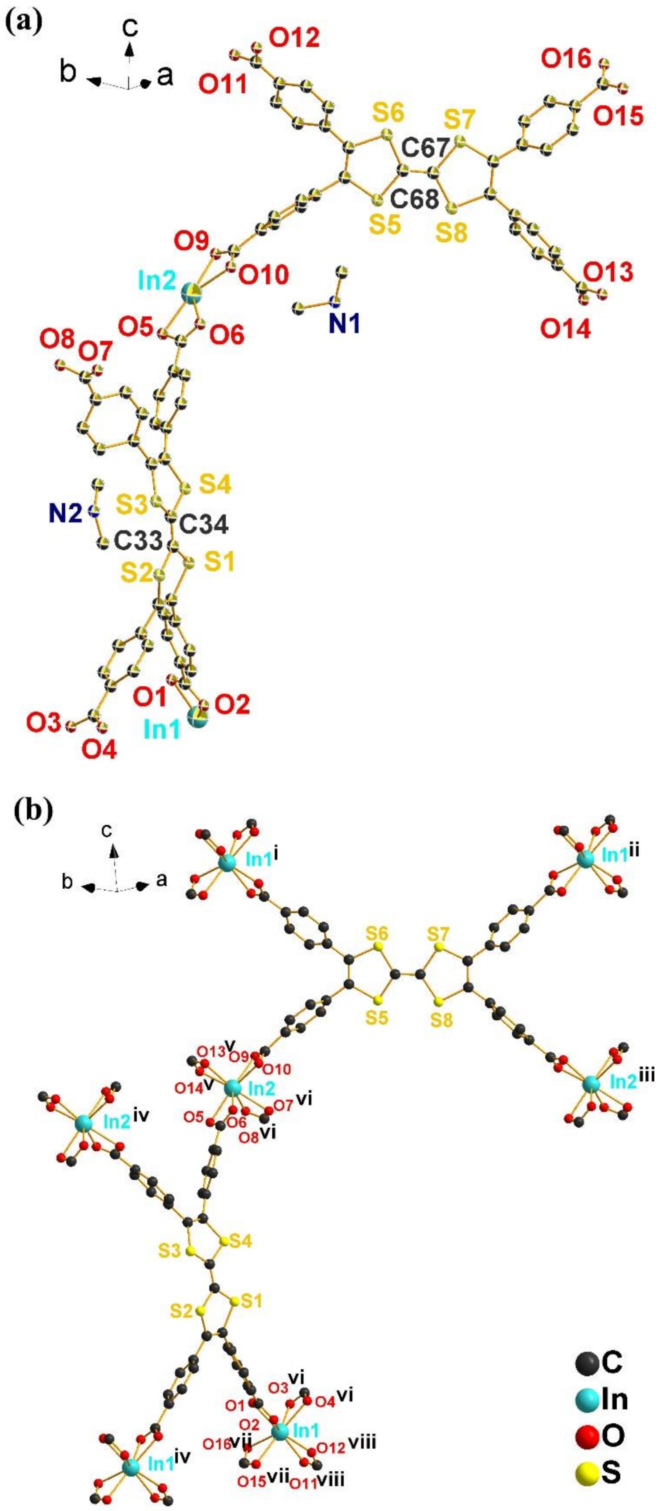


**Supplementary Figure 3.** (a) The asymmetric unit of compound **2**. Displacement ellipsoids are drawn at the 50% probability level. H atoms and labels of benzene carbons are omitted for clarity. (b) The coordination environments of two $TTFTB^{4-}$ ligands and $In^{3+}$ ions in compound **2**. Symmetry transformations used to generate equivalent atoms: i (x, y, 1+z), ii (1+x, -1+y, 1+z), iii (1+x, -1+y, z), iv (-1+x, y, z), v (-1+x, 1+y, z), vi (1+x, y, z), vii (-1+x, 1+y, -1+z), viii (x, y, -1+z).

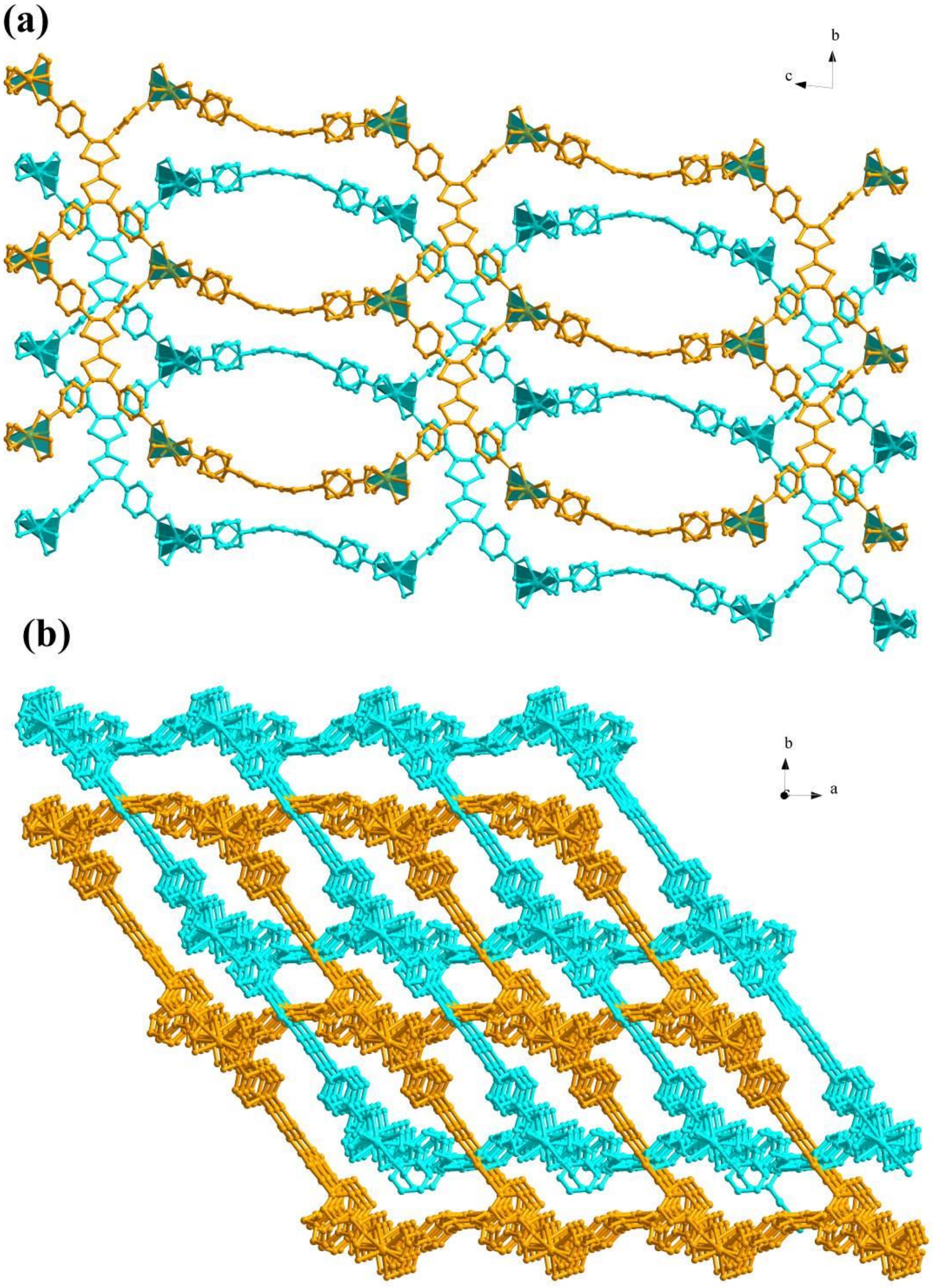


**Supplementary Figure 4.** The 2-fold interpenetrating 3-D framework of compound **2** view in the ***a*** (a) and ***c*** (b) direction.

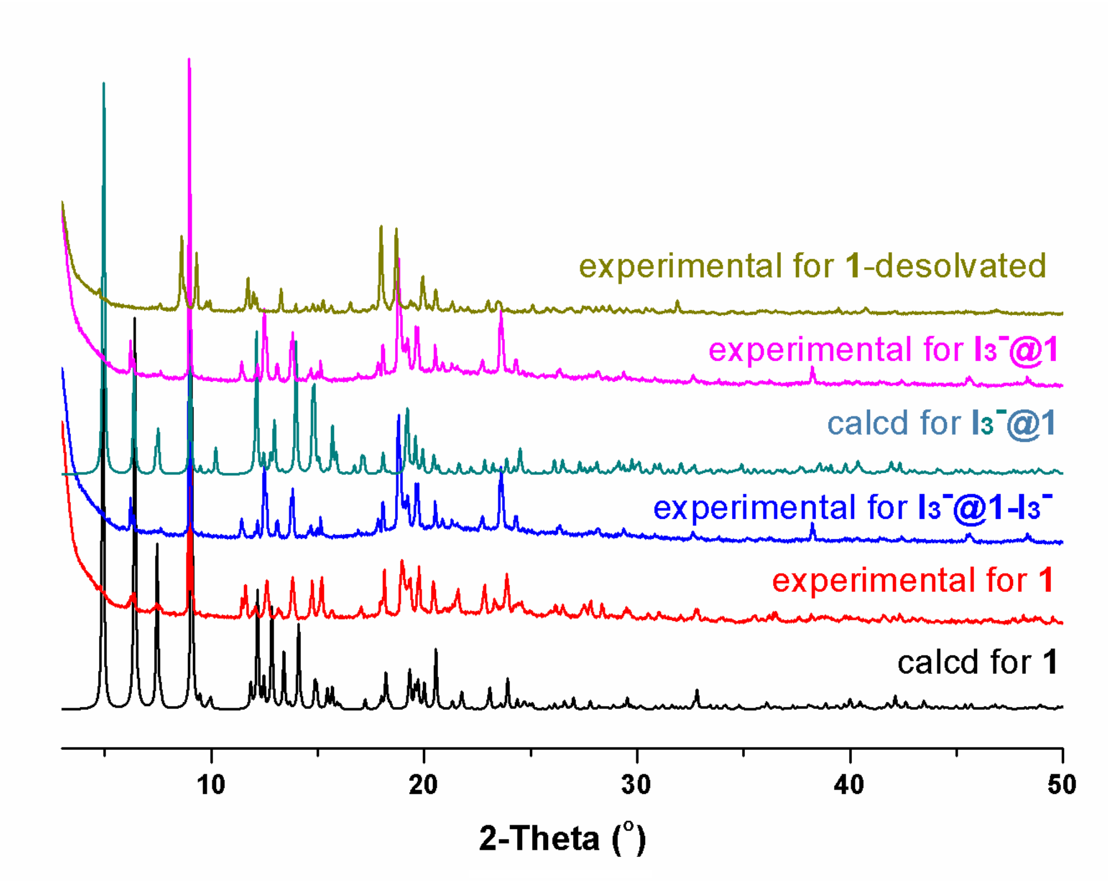


**Supplementary Figure 5.** X-ray powder diffraction patterns of compounds **1**, $I_3^-$@**1** and $I_3^-$@**2**.

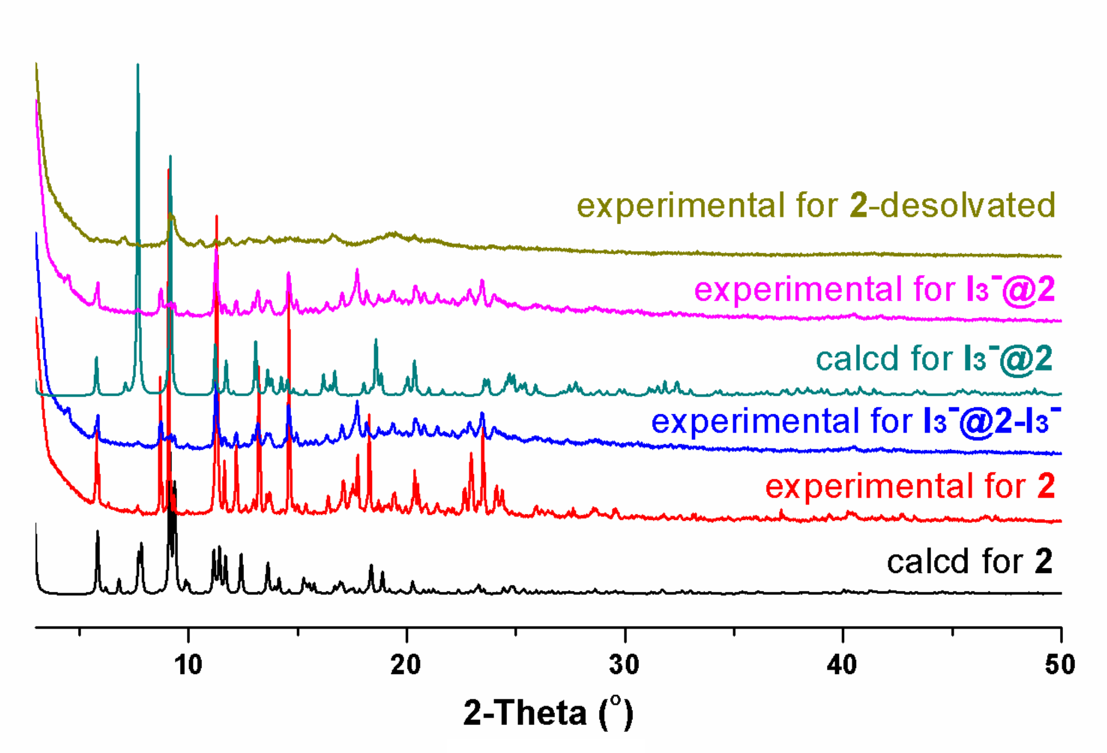


**Supplementary Figure 6.** X-ray powder diffraction patterns of compounds **2**, $I_3^-$@**2** and $I_3^-$@**2.**

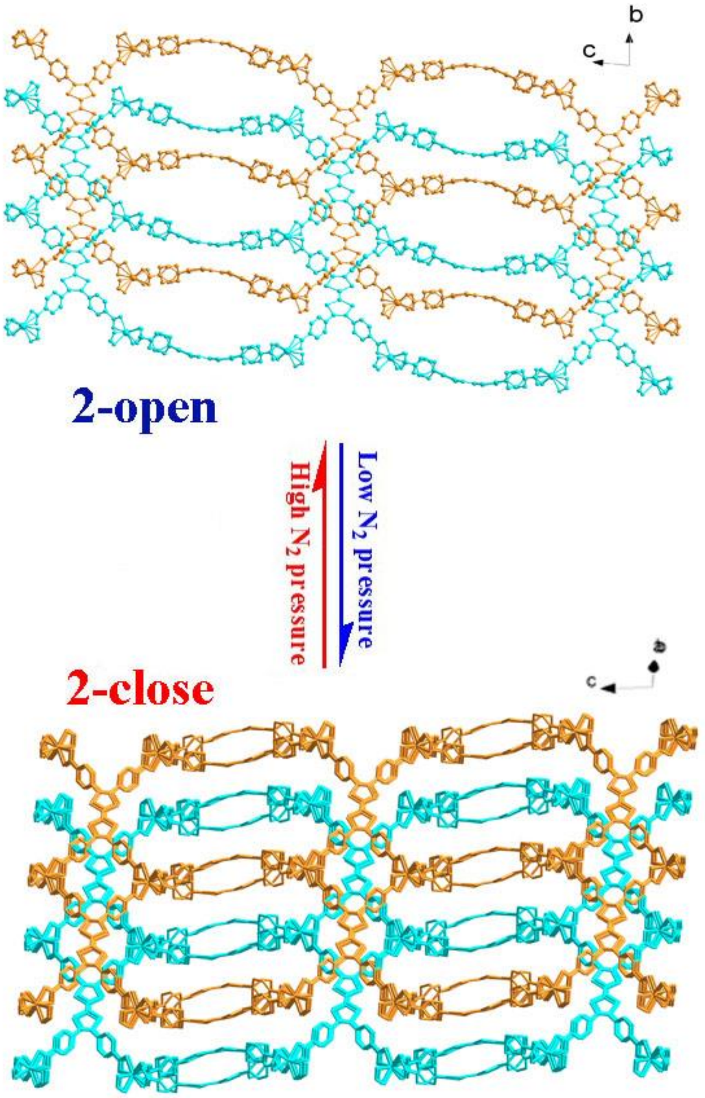


**Supplementary Figure 7.** X-ray single crystal structures of compounds **2-open** and **2-close.**

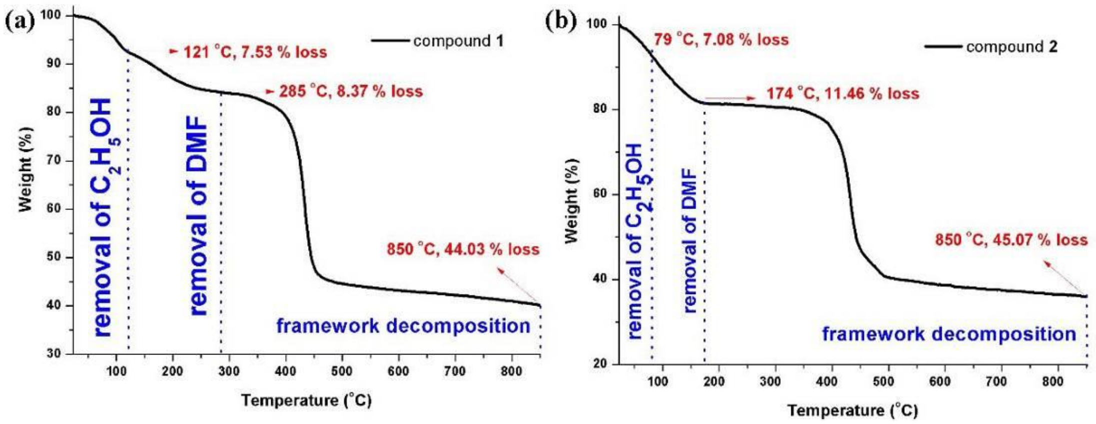


**Supplementary Figure 8.** The TG plots of compounds **1** (a) and **2** (b) at $N_2$ atmosphere. The TGA curve shows nearly three-stage weight loss behavior. In **1**, the first step commences at ambient temperature and completes at 121 ℃, which corresponds exactly to the nominal mass fraction of 0.7 $C_2H_5OH$ molecules (cal: 7.53%). The second loss from 121 ℃ to 285 ℃ corresponds to one DMF molecule (cal: 8.37%). In this stage, compound **1** lost all its guest solvent. At the last stage, along with the removal of $NH_2Me_2^+$, the framework undergoes a gradual collapse. The crystallinity is lost completely and an unknown amorphous product is generated. On the other hand, compound **2** displayed a weight loss at ~79 ℃, indicating desolvation of two $C_2H_5OH$ molecules (cal: 7.02%). The step from 79 ℃ to 174 ℃ corresponds to two DMF molecules (cal: 11.13%). Further heating established that compound **2** was stable up to ~400 ℃

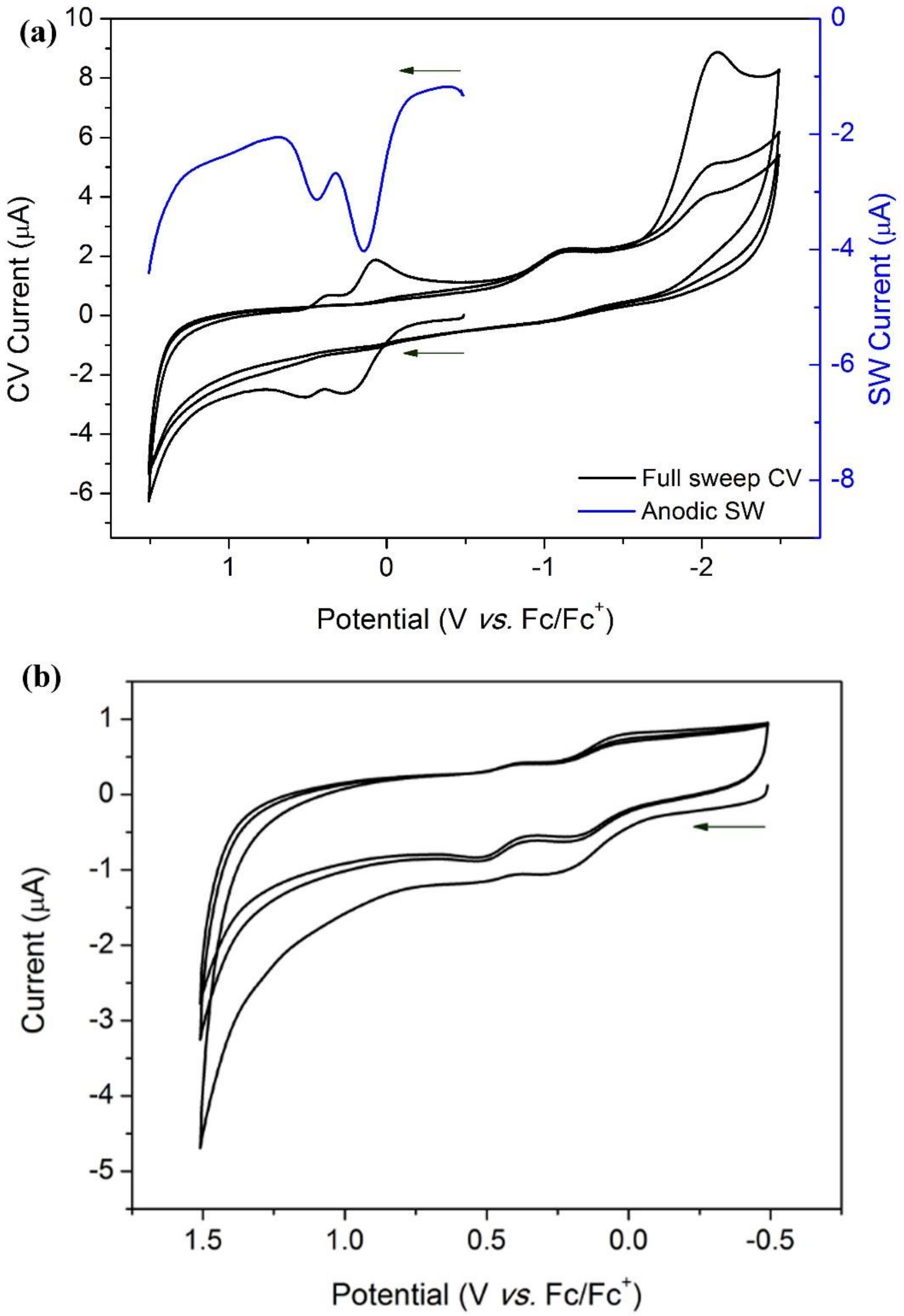


**Supplementary Figure 9.** (a) Solid state CV (black) and SW (blue) voltammograms of **1**. CV was scanned at 100 $mVs^{-1}$ over 2 cycles and SW voltammogram was obtained at 80 mV amplitude and 9 Hz frequency. (b) Solid state CV of **1** in the range -0.49 to 1.51 V vs. $Fc/Fc^{+}$ over 3 consecutive cycles. All the experiments were performed in 0.1 M $LiBF_4$ in $CH_3CN$ supporting electrolyte. Arrow indicates the direction of forward scan.

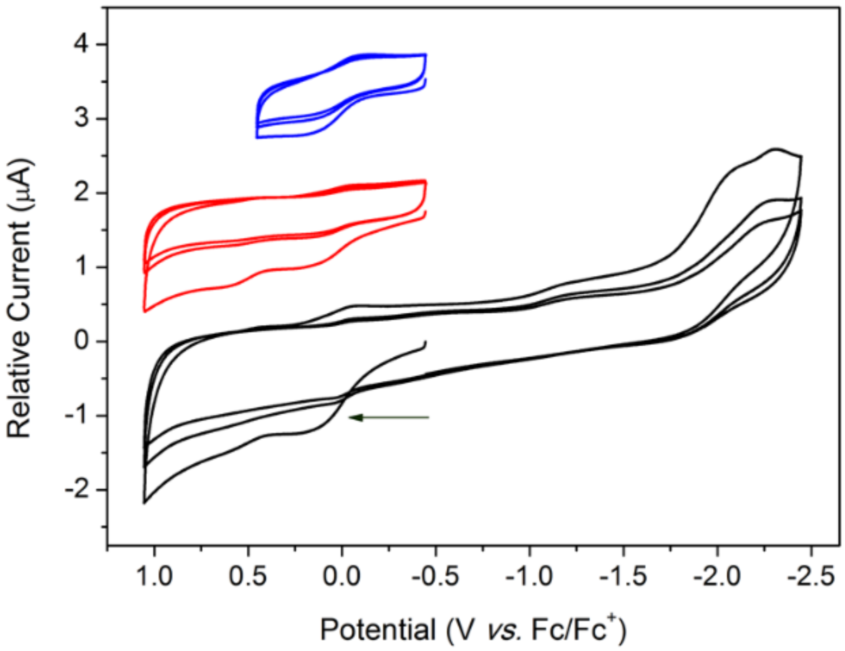


**Supplementary Figure 10.** Solid state cyclic voltammograms of **2** over 3 consecutive cycles and various potential ranges: -2.45-1.05 (black), -0.44-1.05 (red) and -0.45-0.45 (blue) V vs. $Fc/Fc^{+}$.

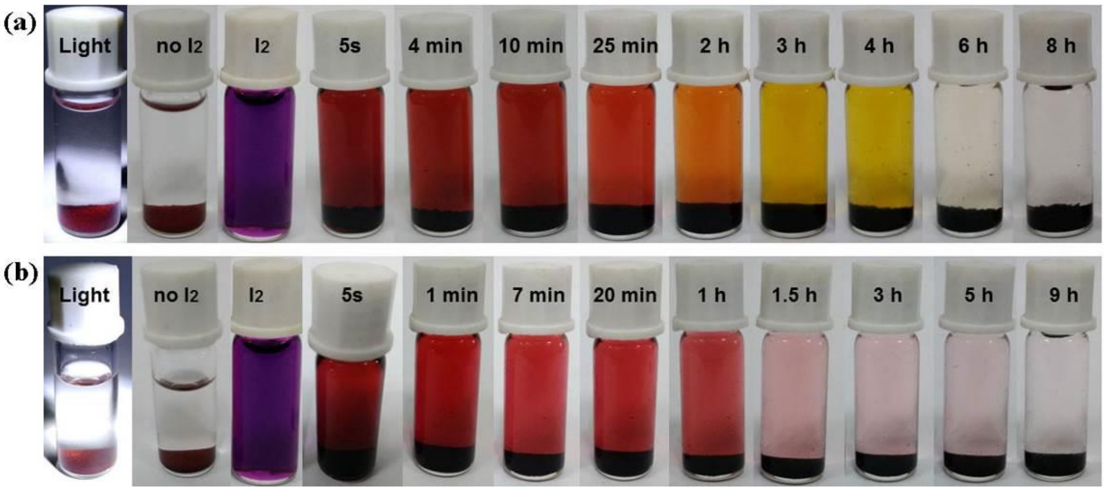


**Supplementary Figure 11.** Photographs showing the visual color change of $I_2$ enrichment progress when crystals of (a)**1** and (b)**2** were soaked in a cyclohexane solution of $I_2$.

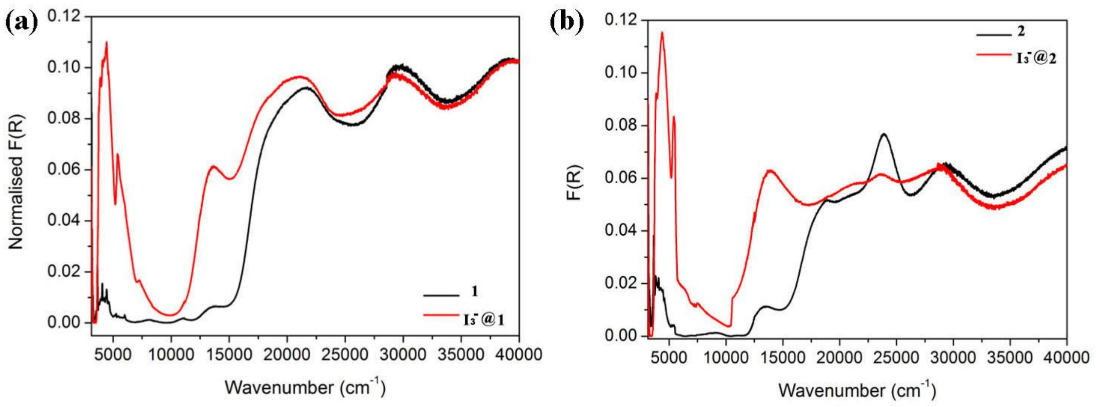


**Supplementary Figure 12.** The diffuse reflectance UV-vis-NIR spectrum of **1**, $I_3^-$@**1, 2** and $I_3^-$@**2**.

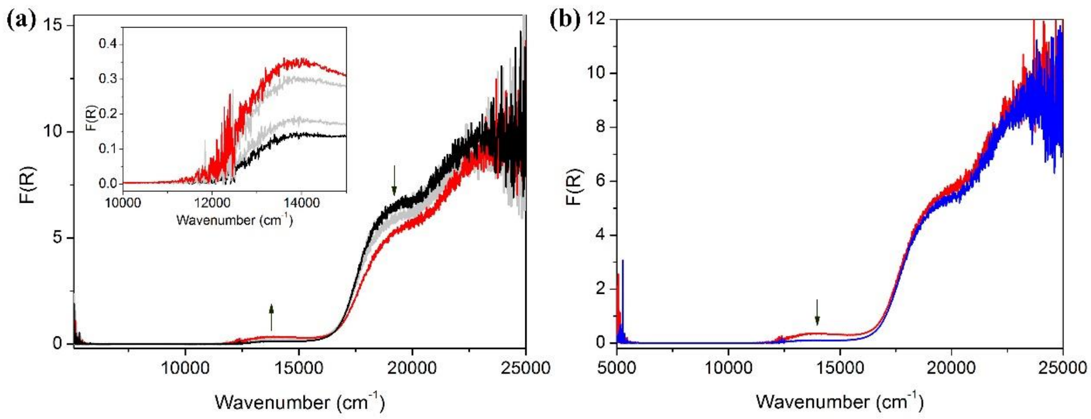


**Supplementary Figure 13.** (a) The Solid state vis-NIR spectroelectrochemistry of compound **1** showing the progression of spectral change from 0 V (black) to 2 V (red). (b) Solid state vis-NIR spectroelectrochemistry of compound **1** showing the progression of spectral change from 2 V (red) back to 0 V (blue). Experiment was performed in 0.1 M $TBAPF_6$ in $CH_3CN$ supporting electrolyte. Arrows indicate the direction of spectral change.

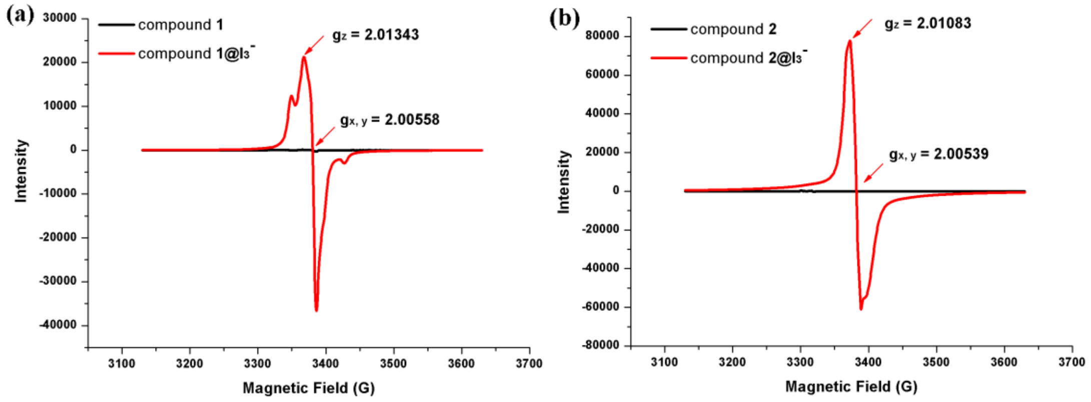


**Supplementary Figure 14.** The Solid state EPR spectrum of (a) **1** and $I_3^-$@**1**, and (b) **2** and $I_3^-$@**2** at 110 K.

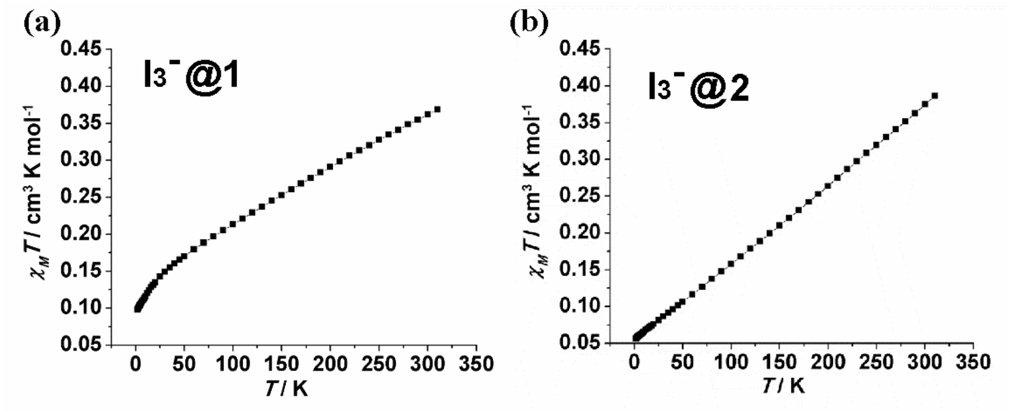


**Supplementary Figure 15.** Magnetic properties of $I_3^-$@**1** (a) and $I_3^-$@**2** (b) in the form of $\chi_M$T versus T.

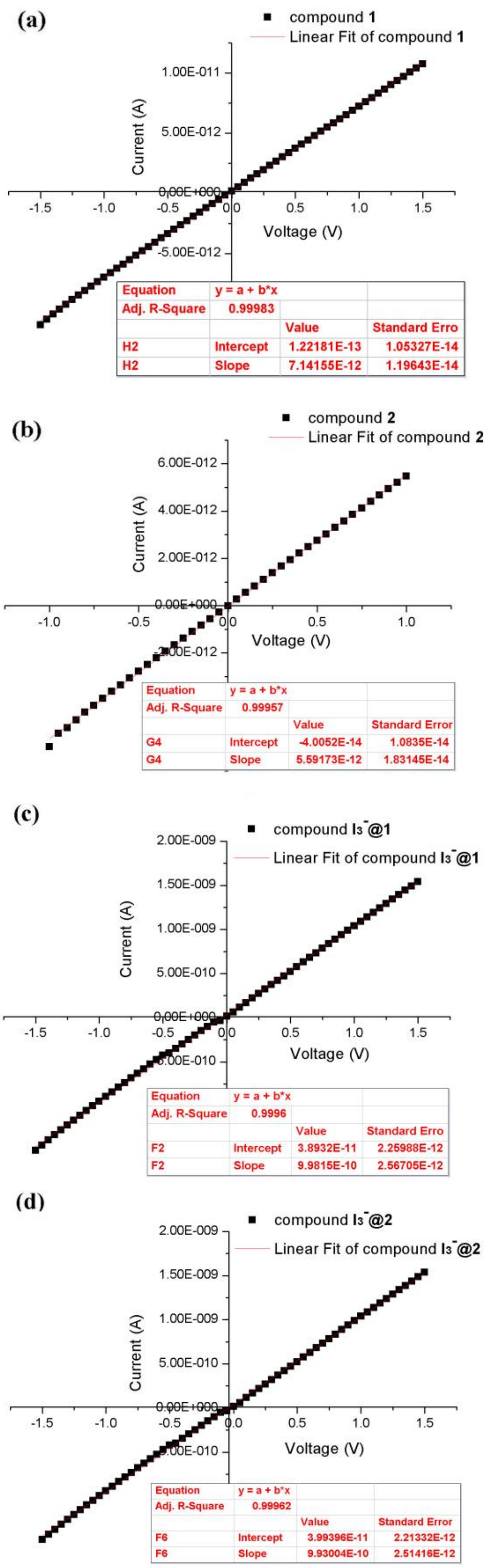


**Supplementary Figure 16.** I-V curves of **1** (a), **2** (b), $I_3^-$@**1** (c) and $I_3^-$@**2** (d).

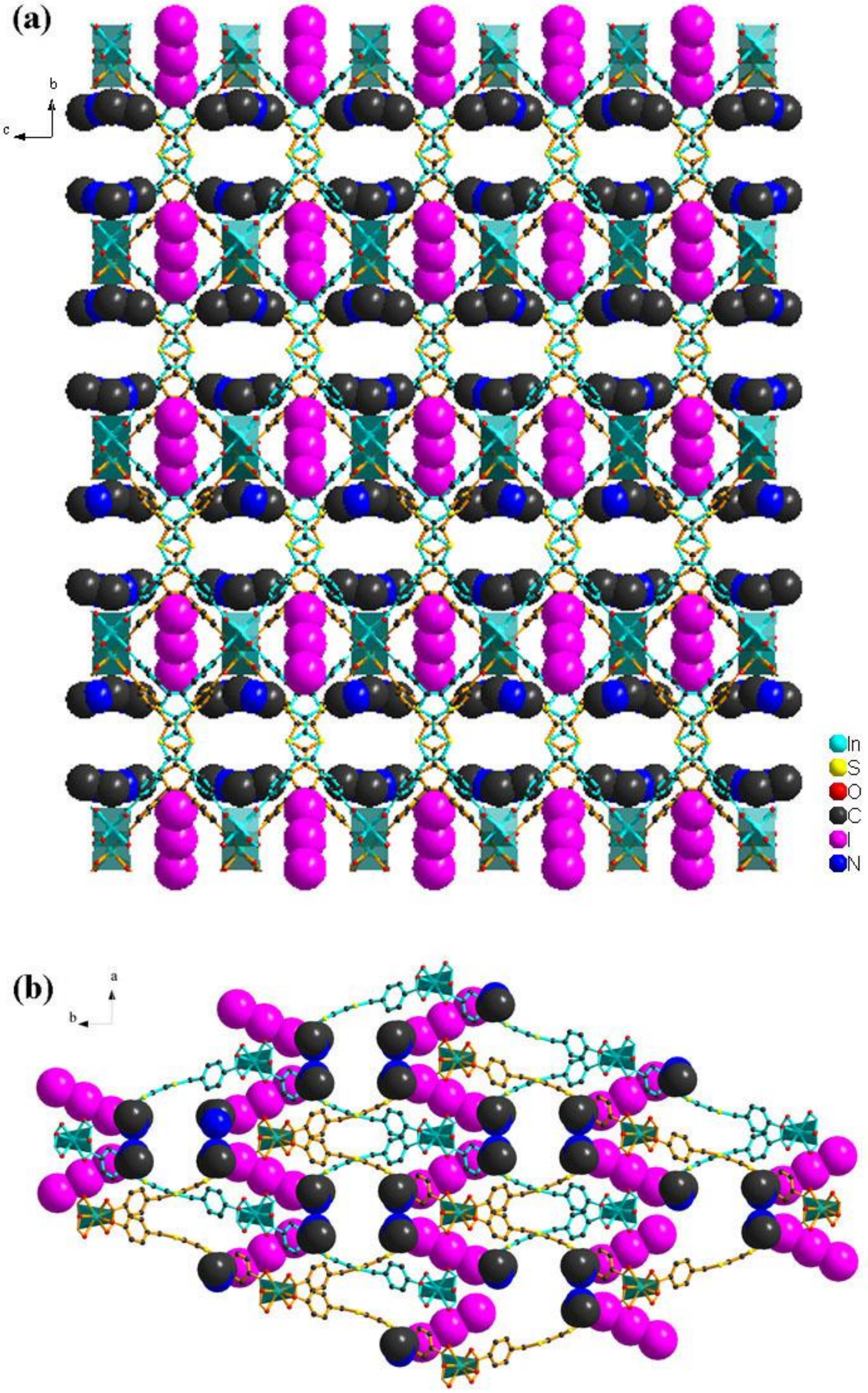


**Supplementary Figure 17.** The 3-D framework of compound $I_3^-$**@1** view in the ***a*** (a) and ***c*** (b) direction.

(a)

(b)

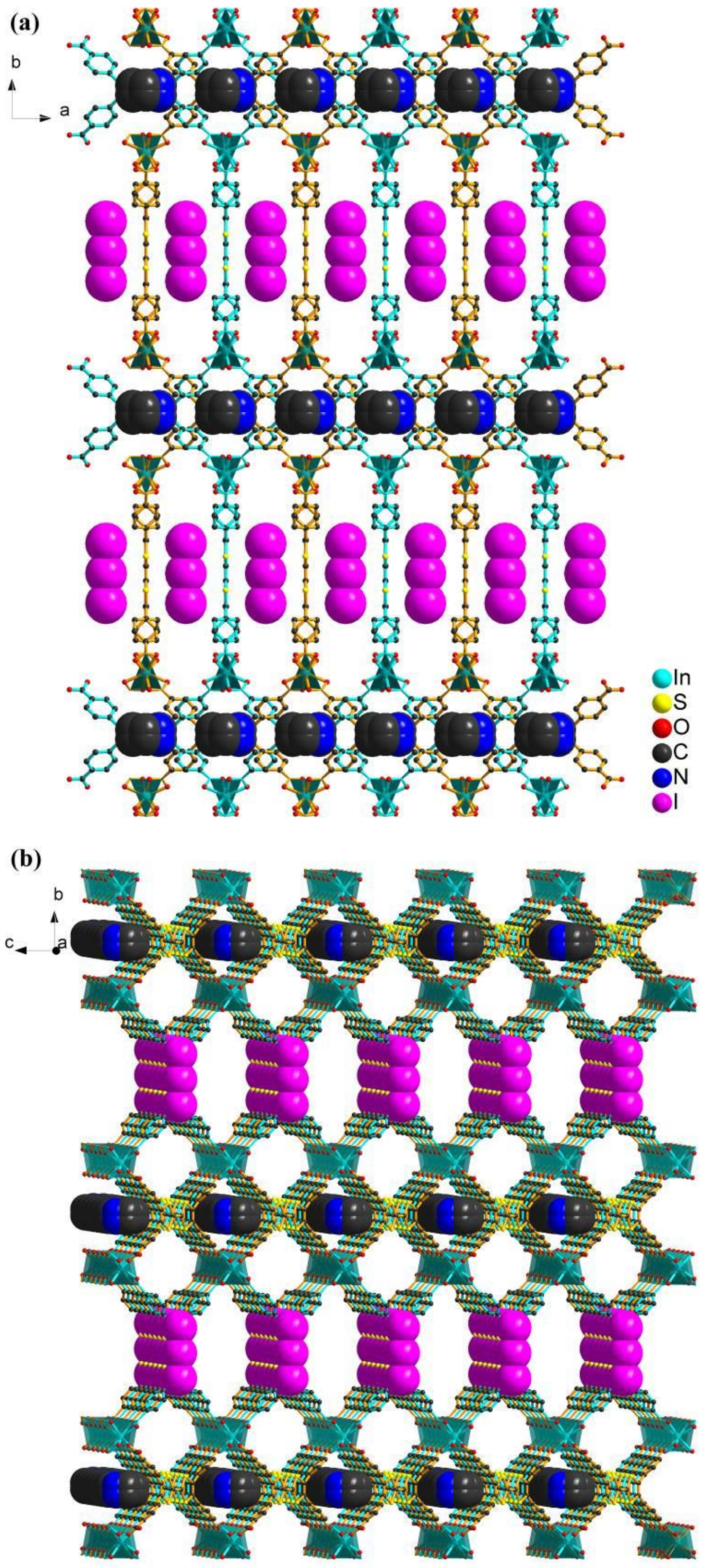


**Supplementary Figure 18.** The 3-D framework of compound $I_3^-$**@2** view in the ***c*** (a) and ***a*** (b) direction.

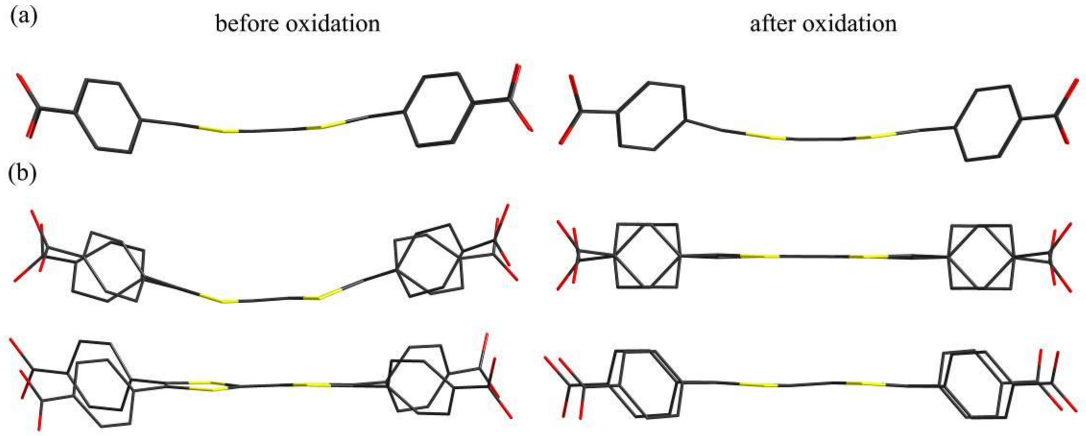


**Supplementary Figure 19.** Conformations of the **TTFTB$^{4-}$** ligand in compounds **1**, $I_3^-$**@1** (a), **2** and $I_3^-$**@2** (b). Hydrogen atoms have been removed for clarity.

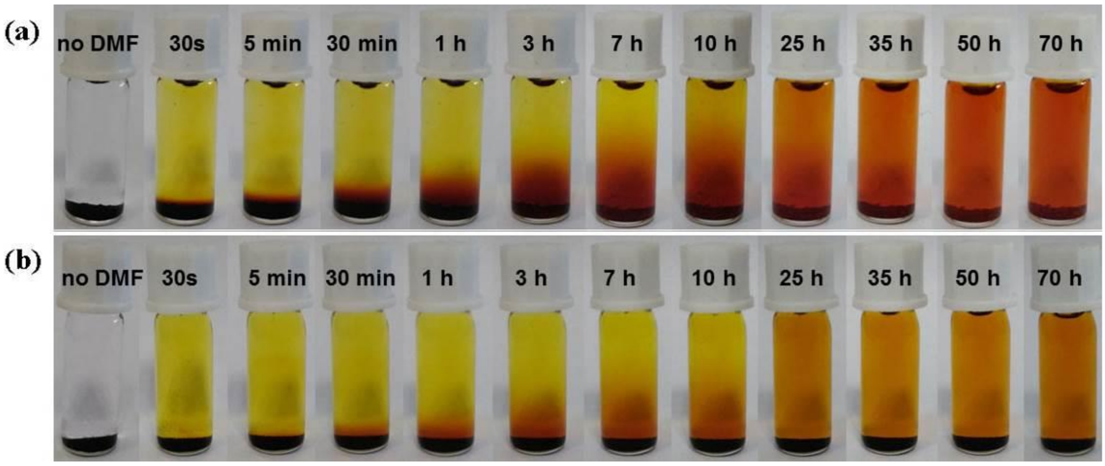


**Supplementary Figure 20.** Photographs showing the visual color change of $I_2$ release progress when crystals of $I_3^-$**@1** (a) and $I_3^-$**@2** (b) were soaked in DMF.

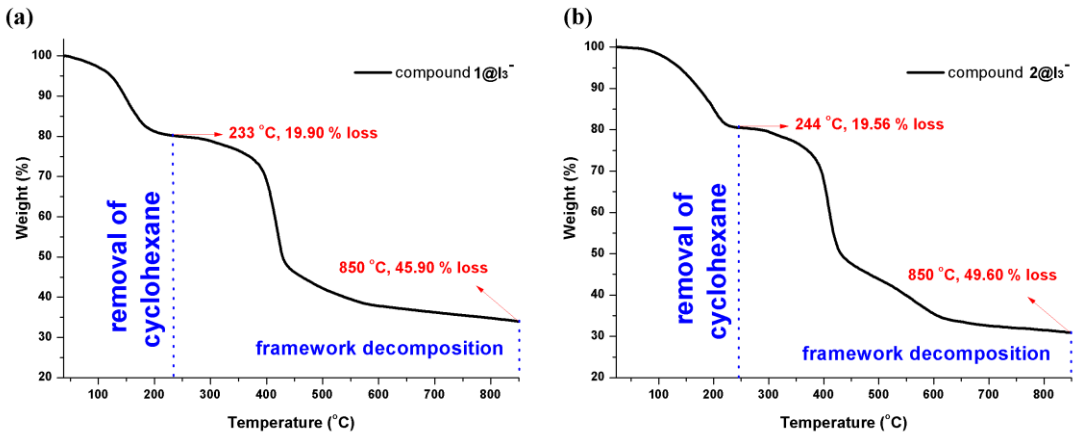


**Supplementary Figure 21.** The TG plots of compounds $I_3^-$**@1** and $I_3^-$**@2** at $N_2$ atmosphere. In $I_3^-$**@1**, the initial weight loss of 19.90% from 23–233 ℃ corresponds to the loss of four cyclohexane molecules (calcd. 19.28%). After the loss of solvent molecules, compound $I_3^-$**@1** is thermally stable until 350 ℃. After that, along with the removal of $NH_2Me_2^+$ and $I_3^-$, the framework gradually collapses. The crystallinity is lost completely and an unknown amorphous product is generated. On the other hand, compound $I_3^-$**@2**, similar to $I_3^-$**@1**, displaying a weight loss at ~244 ℃ indicating desolvation of four cyclohexane molecules (cal: 19.28%). This is followed by a gradual collapse of the framework.

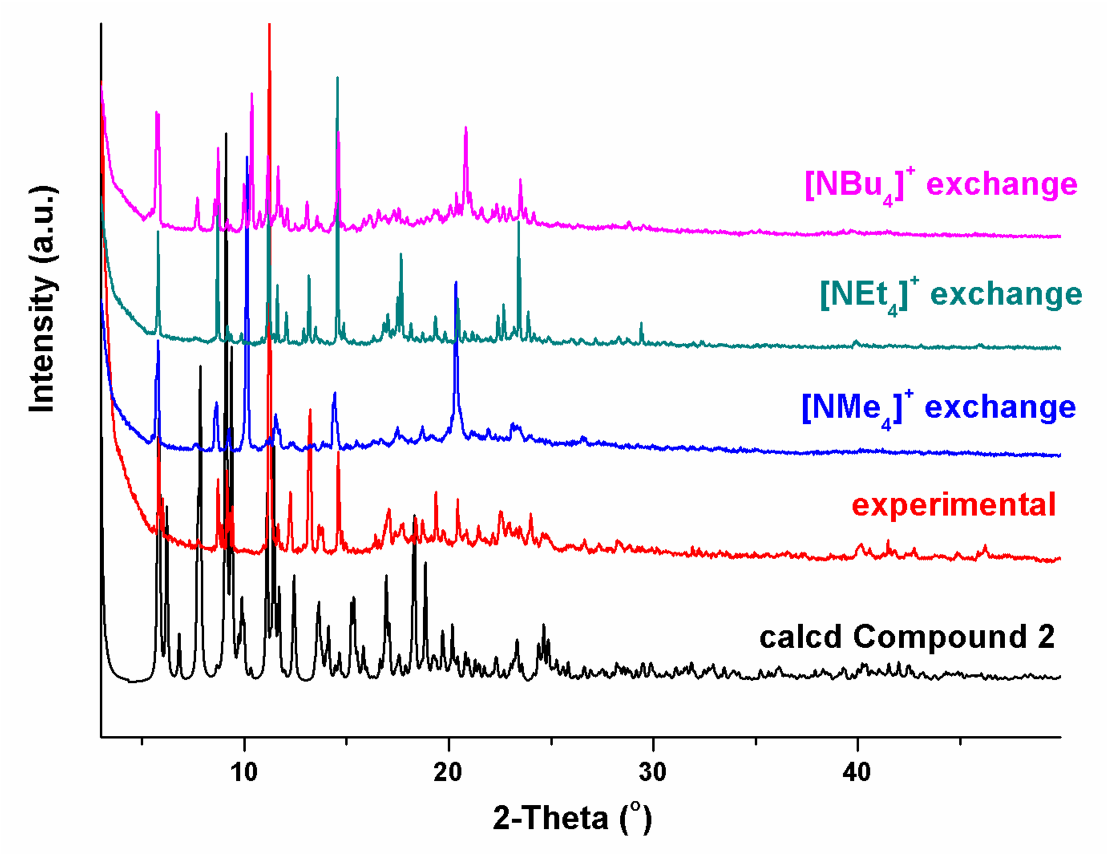


**Supplementary Figure 22.** X-ray powder diffraction patterns of compounds **2**, **[NMe₄]⁺@2**, **[NEt₄]⁺@2** and **[NBu₄]⁺@2**.

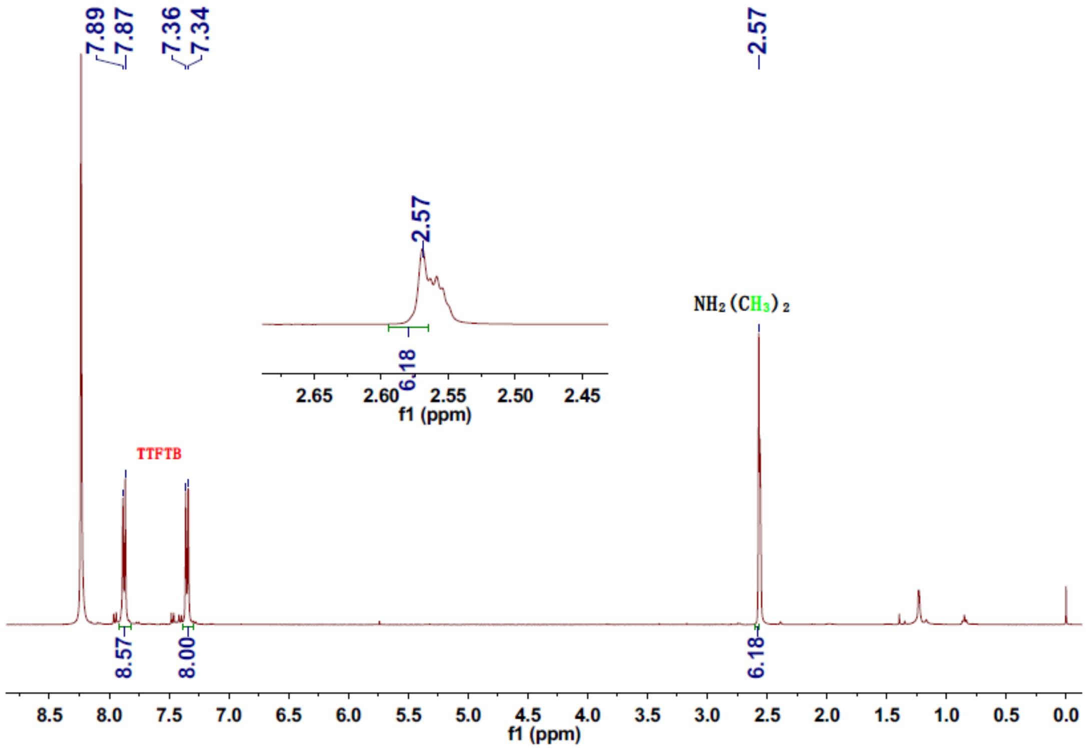


**Supplementary Figure 23.** $^1$H-NMR spectra of $D_2SO_4$ dissolved compound **2** in $d_6$-DMSO.

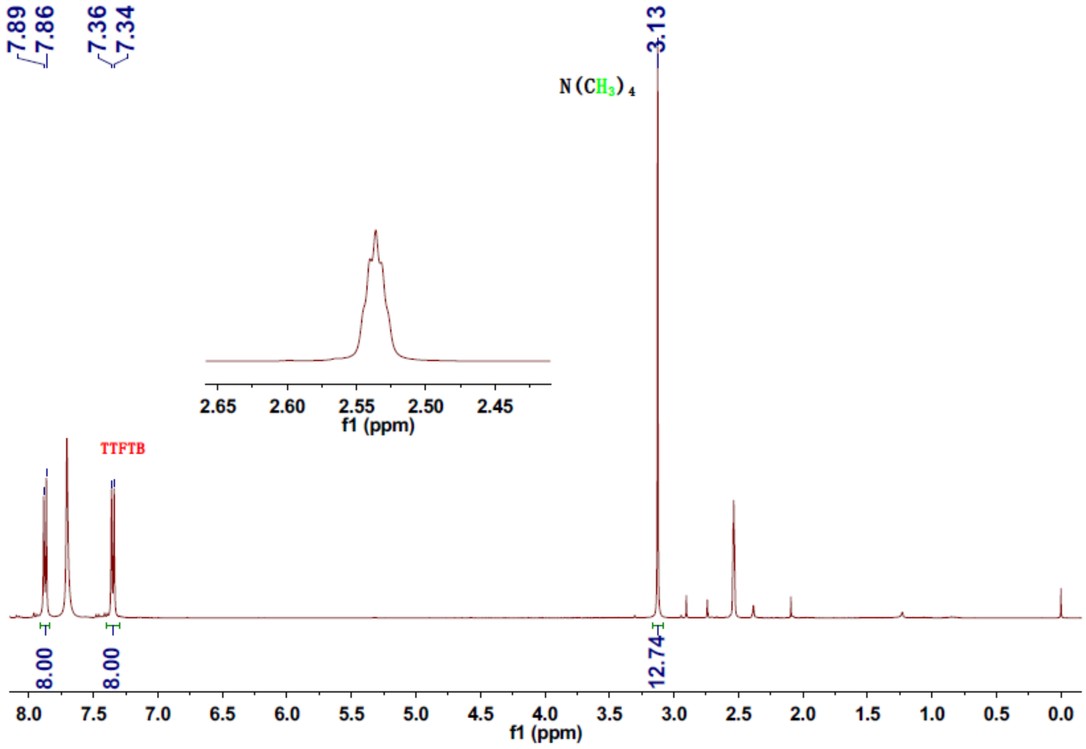


**Supplementary Figure 24.** $^{1}$H-NMR spectra of $D_2SO_4$ dissolved compound **$[NMe_4]^+$@2** in $d_6$-DMSO.

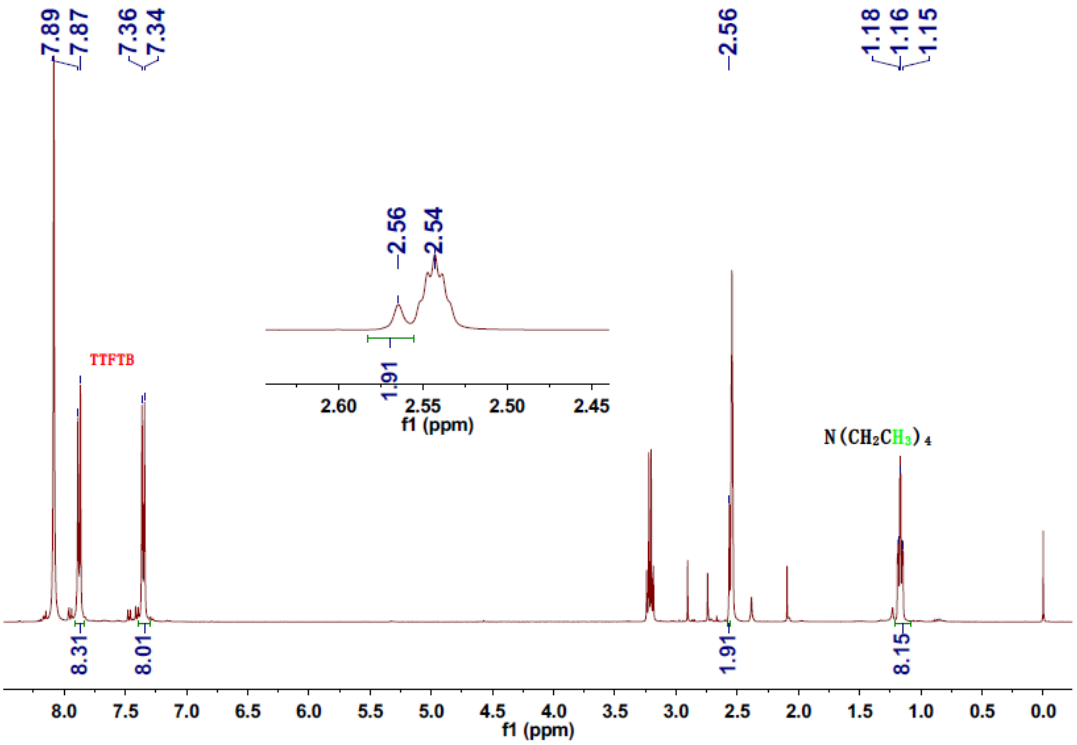


**Supplementary Figure 25.** $^{1}$H-NMR spectra of $D_2SO_4$ dissolved compound **$[NEt_4]^+$@2** in $d_6$-DMSO.

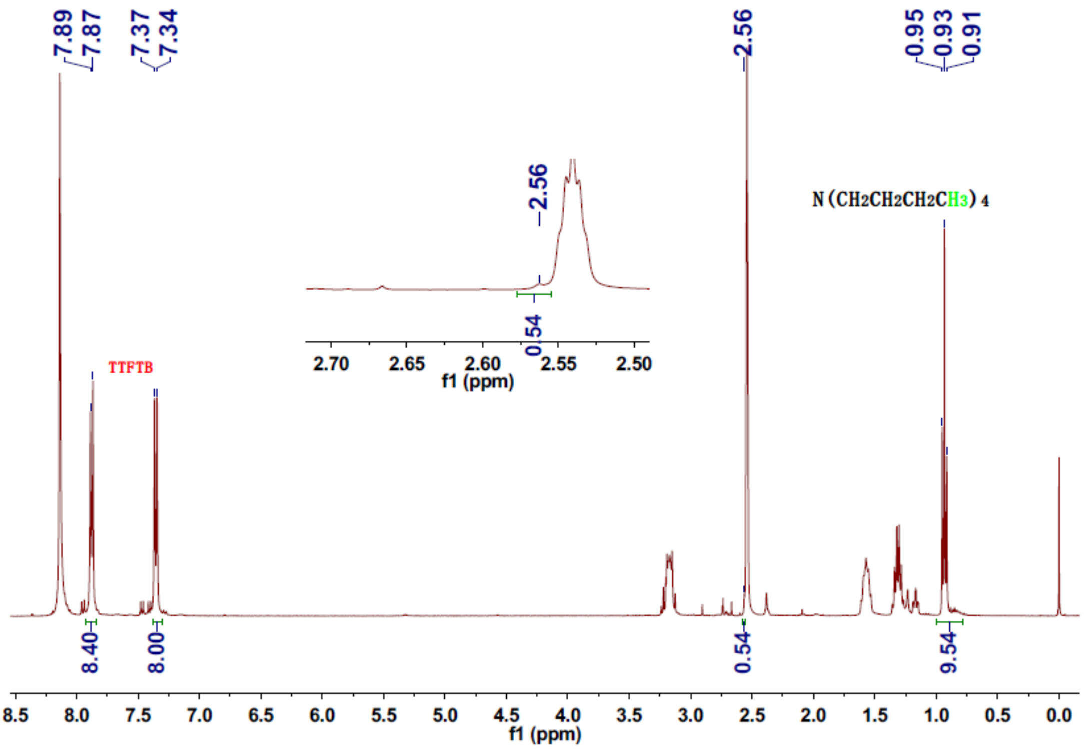


**Supplementary Figure 26.** $^1$H-NMR spectra of $D_2SO_4$ dissolved compound **$[NBu_4]^+$@2** in $d_6$-DMSO.

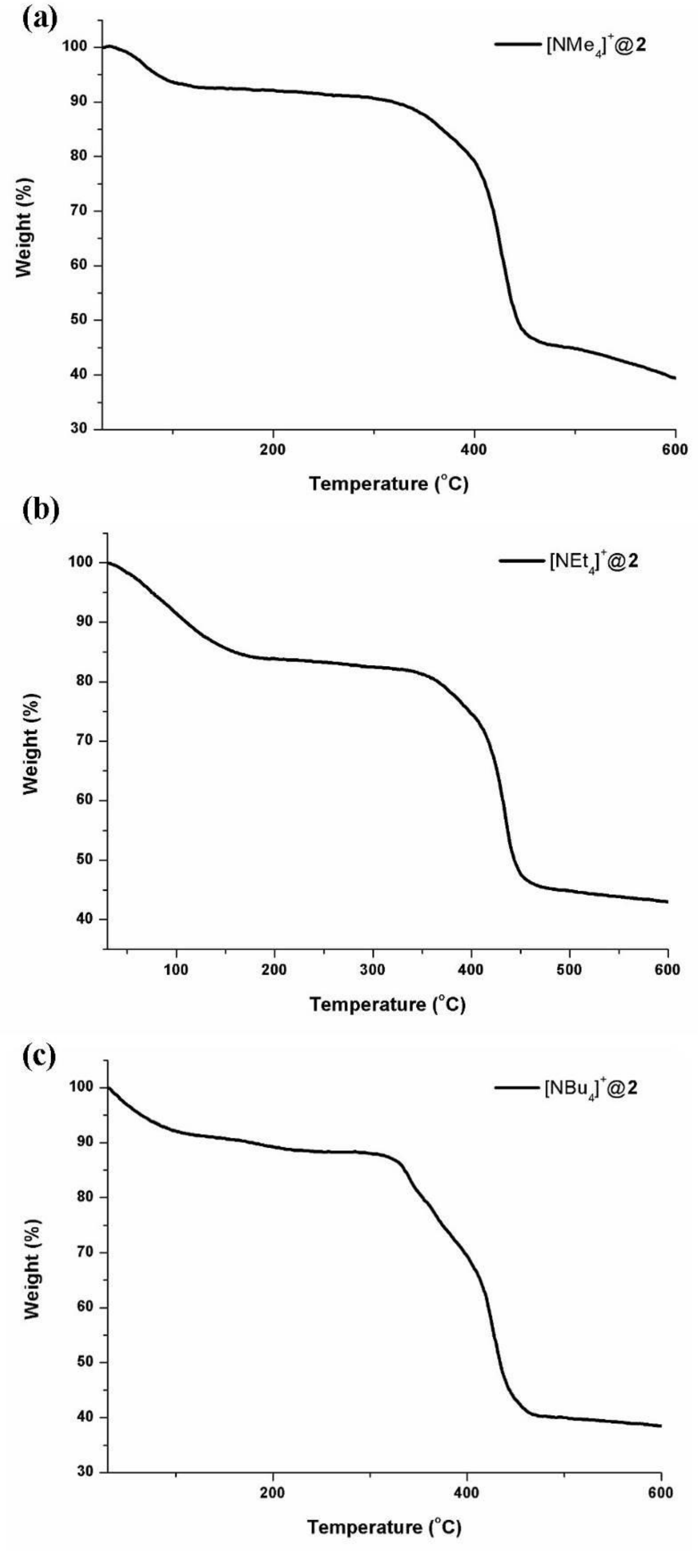


**Supplementary Figure 27.** The TG plots of $[NMe_4]^+$@**2**, $[NEt_4]^+$@**2** and $[NBu_4]^+$@**2** at $N_2$ atmosphere.

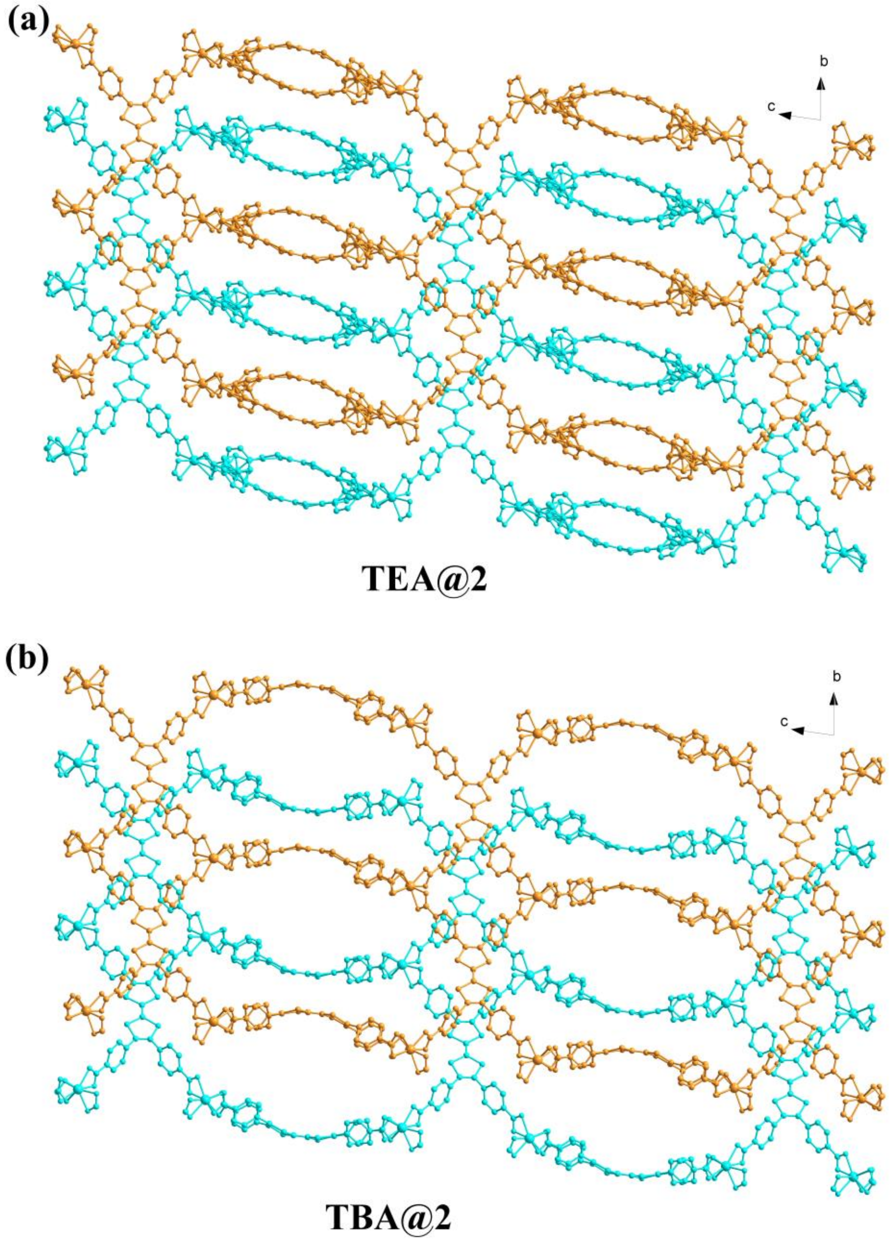


**Supplementary Figure 28.** The 3-D framework of compound $[NEt_4]^+$@**2** (a) and $[NBu_4]^+$@**2** (b) viewed from the ***a*** direction.

**Table S1** Crystal date and structure refinement parameters for compound **1** and $I_3^-$**@1**.

| | **1** | $I_3^-$**@1** |
|---|---|---|
| Empirical formula | $C_{36}H_{24}InNO_8S_4$ | $C_{70}H_{40}In_2NO_{16}S_8I_3$ |
| Formula weight | 841.66 | 2017.85 |
| Temperature (K) | 153 | 153 |
| Crystal system | orthorhombic | orthorhombic |
| Wavelength (Å) | 0.71073 | 0.71073 |
| Space group | *Cccm* (No. 66) | *Cccm* (No. 66) |
| Unit cell dimensions (Å, °) | | |
| a | 14.907(4) | 15.014(2) |
| b | 35.859(9) | 35.599(4) |
| c | 23.193(6) | 23.505(4) |
| Volume ($Å^3$) | 12398(6) | 12563(3) |
| Z | 8 | 4 |
| Calculated density ($gcm^{-3}$) | 0.902 | 1.067 |
| F(000) | 3392 | 3920 |
| Absorption coefficient, $\mu/mm^{-1}$ | 0.546 | 1.276 |
| No. of reflections measured | 44870 | 51625 |
| No. of independent reflections | 5613 | 7030 |
| θ (°) | 1.1-25.0 | 2.1-27.0 |
| $R_{int}$ | 0.049 | 0.007 |
| $R_1$, $wR_2$ [$I \geq 2\sigma$ (I)] | 0.0495/ 0.1454 | 0.0456/ 0.1023 |
| $R_1$, $wR_2$ [all data] | 0.0582/ 0.1582 | 0.0505/ 0.1040 |
| GOF | 1.13 | 1.04 |
| Largest diff. peak and hole($eÅ^{-3}$) | 1.18 / -0.55 | 1.71 / -1.30 |

$^aR1 = \Sigma||F_o| - |F_c||/\Sigma|F_o|$, $wR_2 = [\Sigma w(F_o^2 - F_c^2)^2/\Sigma w(F_o^2)^2]^{1/2}$.

**Table S2** Selected bond lengths (Å) and angles ( °) of compound **1**.

| | | | |
|---|---|---|---|
| C(9)-C(10) | 1.343(9) | | |
| In(1)-O(1) | 2.226(3) | In(1)-O(2) | 2.324(3) |
| In(1)-O(3)#1 | 2.283(3) | In(1)-O(4) #1 | 2.248(4) |
| O(1) -In(1)-O(2) | 57.13(9) | O(1) -In(1)-O(1) #2 | 89.93(1) |
| O(1) -In(1)-O(2) #2 | 82.46(9) | O(1) -In(1)-O(3) #1 | 86.32(1) |
| O(1) -In(1)-O(4) #1 | 91.68(1) | O(1) -In(1)-O(3)#3 | 139.73(1) |
| O(1) -In(1)-O(4)#3 | 163.60(1) | O(2) -In(1)-O(2)#2 | 123.11(9) |
| O(2) -In(1)-O(3) #1 | 82.65(1) | O(2) -In(1)-O(4) #1 | 84.73(1) |
| O(2) -In(1)-O(3)#3 | 79.5(2) | O(2) -In(1)-O(4) #3 | 139.24(1) |
| O(3)#1-In(1)-O(4)#1 | 56.67(1) | O(3) #1-In(1)-O(3) #3 | 120.82(2) |
| O(3) #1-In(1)-O(4) #3 | 81.92(2) | O(4) #1-In(1)-O(4) #3 | 91.36(3) |

Symmetry transformations used to generate equivalent atoms:

#1 -1/2+x, 1/2+y, 1-z; #2 -x, y, 3/2-z; #3 1/2-x, 1/2+y, 1/2+z.

**Table S3** Selected bond lengths (Å) and angles (°) of compound $I_3^-$@**1**.

| | | | |
|---|---|---|---|
| I(1)-I(2) | 3.0235(8) | C(9)-C(10) | 1.387(6) |
| In(1)-O(1) | 2.364(2) | In(1)-O(2) | 2.214(2) |
| In(1)-O(3)#1 | 2.215(2) | In(1)-O(4) #1 | 2.310(2) |
| O(1) -In(1)-O(2) | 56.80(7) | O(1) -In(1)-O(1) #2 | 124.46(8) |
| O(1) -In(1)-O(2) #2 | 83.46(7) | O(1) -In(1)-O(3) #1 | 84.70(8) |
| O(1) -In(1)-O(4) #1 | 125.30(7) | O(1) -In(1)-O(3)#3 | 140.11(8) |
| O(1) -In(1)-O(4)#3 | 82.23(7) | O(2) -In(1)-O(2)#2 | 89.3(3) |
| O(2) -In(1)-O(3) #1 | 94.13(8) | O(2) -In(1)-O(4) #1 | 85.37(7) |
| O(2) -In(1)-O(3)#3 | 163.00(8) | O(2) -In(1)-O(4) #3 | 139.03(7) |
| O(3)#1-In(1)-O(4)#1 | 57.92(8) | O(3) #1-In(1)-O(3) #3 | 87.18(8) |
| O(3) #1-In(1)-O(4) #3 | 80.97(8) | O(4) #1-In(1)-O(4) #3 | 123.28(8) |

Symmetry transformations used to generate equivalent atoms:

#1 -1/2+x, -1/2+y, 1-z; #2 -x, y, 1/2-z; #3 1/2+x, 1/2+y, 1-z.

**Table S4** Crystal date and structure refinement parameters for compound **2** and $I_3^-$@**2**.

| | **2** | $I_3^-$**@2** |
|---|---|---|
| Empirical formula | $C_{72}H_{48}O_{16}In_2N_2S_8$ | $C_{70}H_{40}In_2NO_{16}S_8I_3$ |
| Formula weight | 1683.24 | 2017.85 |
| Temperature (K) | 153 | 153 |
| Crystal system | Triclinic | Monoclinic |
| Wavelength (Å) | 0.71073 | 0.71073 |
| Space group | $P\bar{1}$ (No. 2) | $P2/m$ (No. 10) |
| Unit cell dimensions (Å, °) | | |
| a | 12.247(3) | 7.549(2) |
| b | 15.245(4) | 30.567(7) |
| c | 30.785(3) | 12.423(3) |
| α | 83.165(2) | 90 |
| β | 88.424(3) | 90.801(6) |
| γ | 88.289(2) | 90 |
| Volume (Å$^3$) | 5703(2) | 2866.4(2) |
| Z | 2 | 1 |
| Calculated density (gcm$^{-3}$) | 0.980 | 1.169 |
| F(000) | 1696 | 980 |
| Absorption coefficient, $\mu$/mm$^{-1}$ | 0.594 | 1.398 |
| No. of reflections measured | 45598 | 19666 |
| No. of independent reflections | 22564 | 5321 |
| θ (°) | 2.0-27.0 | 2.1-25.3 |
| $R_{int}$ | 0.072 | 0.064 |
| $R_1$, $wR_2$ $[I \geq 2\sigma (I)]$ | 0.0538/ 0.1368 | 0.0548/ 0.1254 |
| $R_1$, $wR_2$ *[all data]* | 0.0743/ 0.1408 | 0.0859/ 0.1294 |
| GOF | 1.02 | 1.09 |
| Largest diff. peak and hole(eÅ$^{-3}$) | 1.15 /-0.94 | 0.81 / -1.03 |

$^a R1 = \Sigma||F_o| - |F_c||/\Sigma|F_o|$, $wR_2 = [\Sigma w(F_o^2 - F_c^2)^2/\Sigma w(F_o^2)^2]^{1/2}$.

**Table S5** Selected bond lengths (Å) and angles (°) of compound **2**.

| | | | |
|---|---|---|---|
| C(33)-C(34) | 1.319(7) | C(67)-C(68) | 1.350(6) |
| In(1)-O(1) | 2.228(3) | In(1)-O(2) | 2.367(3) |
| In(1)-O(3)#1 | 2.287(4) | In(1)-O(4) #1 | 2.239(4) |
| In(1)-O(11)#2 | 2.407(4) | In(1)-O(12)#2 | 2.244(3) |
| In(1)-O(15)#3 | 2.296(3) | In(1)-O(16)#3 | 2.267(3) |
| In(2)-O(5) | 2.212(4) | In(2)-O(6) | 2.304(4) |
| In(2)-O(9) | 2.415(4) | In(2)-O(10) | 2.158(4) |
| In(2)-O(7)#1 | 2.320(5) | In(2)-O(8)#1 | 2.477(4) |
| In(2)-O(13)#4 | 2.288(3) | In(2)-O(14)#4 | 2.235(3) |
| O(1) -In(1)-O(2) | 56.54(13) | O(1) -In(1)-O(3) #1 | 83.76(13) |
| O(1) -In(1)-O(4) #1 | 82.61(15) | O(1) -In(1)-O(11) #2 | 164.80(16) |
| O(1) -In(1)-O(12) #2 | 135.84(13) | O(1) -In(1)-O(15)#3 | 93.76(11) |
| O(1) -In(1)-O(16)#3 | 84.33(13) | O(2) -In(1)-O(3)#1 | 122.08(12) |
| O(2) -In(1)-O(4) #1 | 76.71(15) | O(2) -In(1)-O(11) #2 | 138.37(13) |
| O(2) -In(1)-O(12)#2 | 80.03(10) | O(2) -In(1)-O(15) #3 | 79.02(10) |
| O(2) -In(1)-O(16) #3 | 119.10(10) | O(3) #1-In(1)-O(4) #1 | 56.10(16) |
| O(3) #1-In(1)-O(11) #2 | 57.0(5) | O(3) #1-In(1)-O(12) #2 | 118.9(4) |
| O(3) #1-In(1)-O(15) #3 | 150.36(13) | O(3) #1-In(1)-O(16) #3 | 93.24(13) |
| O(4) #1-In(1)-O(11) #2 | 102.58(15) | O(4) #1-In(1)-O(12) #2 | 79.80(13) |
| O(4) #1-In(1)-O(15) #3 | 153.02(14) | O(4) #1-In(1)-O(16) #3 | 147.73(14) |
| O(11) #2-In(1)-O(12) #2 | 59.36(13) | O(11) #2-In(1)-O(15) #3 | 87.88(11) |
| O(11) #2-In(1)-O(16) #3 | 83.89(13) | O(12) #2-In(1)-O(15) #3 | 84.57(10) |
| O(12) #2-In(1)-O(16) #3 | 128.40(10) | O(15) #3-In(1)-O(16) #3 | 57.15(10) |
| O(5)-In(2)-O(6) | 56.67(15) | O(5)-In(2)-O(7) #1 | 92.60(16) |
| O(5)-In(2)-O(8) #1 | 82.75(13) | O(5)-In(2)-O(9) | 159.86(13) |
| O(5)-In(2)-O(10) | 142.81(16) | O(5) -In(2)-O(13)#4 | 92.66(12) |
| O(5) -In(2)-O(14)#4 | 88.68(14) | O(6)-In(2)-O(7)#1 | 81.29(15) |
| O(6)-In(2)-O(8)#1 | 117.25(13) | O(6)-In(2)-O(9) | 141.23(13) |
| O(6)-In(2)-O(10) | 86.14(14) | O(6) -In(2)-O(13)#4 | 83.02(12) |

| | | | |
|---|---|---|---|
| O(6) -In(2)-O(14)#4 | 127.37(12) | O(7)#1-In(2)-O(8) #1 | 52.52(16) |
| O(7)#1-In(2)-O(9) | 83.69(14) | O(7)#1-In(2)-O(10) | 80.90(15) |
| O(7) #1-In(2)-O(13)#4 | 157.06(14) | O(7) #1-In(2)-O(14)#4 | 144.31(14) |
| O(8)#1-In(2)-O(9) | 79.30(12) | O(8)#1-In(2)-O(10)#4 | 119.10(14) |
| O(8)#1-In(2)-O(13) #4 | 150.40(13) | O(8)#1-In(1)-O(14) #4 | 92.43(13) |
| O(9)-In(2)-O(10) | 56.20(13) | O(9) -In(2)-O(13) #4 | 98.45(11) |
| O(9) -In(2)-O(14)#4 | 83.08(10) | O(10)-In(2)-O(13) #4 | 81.44(12) |
| O(10)-In(2)-O(14)#4 | 117.57(13) | O(13)#4-In(2)-O(14)#4 | 58.14(11) |

Symmetry transformations used to generate equivalent atoms:

#1 1+x, y, z; #2 x, y, -1+z; #3 -1+x, 1+y, -1+z; #4 -1+x, 1+y, z.

**Table S6** Selected bond lengths (Å) and angles (°) of compound $I_3^-$@**2**.

| | | | |
|---|---|---|---|
| I(1)-I(2) | 2.9910(9) | | |
| C(9)-C(9) #1 | 1.418(13) | C(18)-C(18) #2 | 1.416(11) |
| In(1)-O(1) | 2.307(4) | In(1)-O(2) | 2.271(5) |
| In(1)-O(3) | 2.228(4) | In(1)-O(4) | 2.241(4) |
| O(1) -In(1)-O(2) | 53.82(18) | O(1) -In(1)-O(3) | 86.94(16) |
| O(1) -In(1)-O(4) | 144.58(17) | O(1)#3-In(1)-O(2) | 79.06(17) |
| O(1) -In(1)-O(2)#3 | 79.06(17) | O(1) -In(1)-O(3)#3 | 117.70(15) |
| O(1) -In(1)-O(4)#3 | 85.57(15) | O(2) -In(1)-O(3) | 139.66(17) |
| O(2) -In(1)-O(4) | 161.29(17) | O(2)#3-In(1)-O(3) | 80.10(16) |
| O(2) -In(1)-O(2) #3 | 83.16(18) | O(2) -In(1)-O(3)#3 | 80.10(16) |
| O(2) -In(1)-O(4)#3 | 96.38(16) | O(3) -In(1)-O(4) | 57.80(15) |
| O(3) -In(1)-O(1)#3 | 117.70(15) | O(3) -In(1)-O(2)#3 | 80.10(16) |
| O(3)-In(1)-O(4)#3 | 88.55(15) | O(3) -In(1)-O(3)#3 | 133.52(15) |

Symmetry transformations used to generate equivalent atoms:

#1 1-x,1-y,2-z; #2 -1-x,y,-z; #31-x, y, 1-z.

**Table S7** The shape parameters of **1**, $I_3^-$**@1**, **2** and $I_3^-$**@2** used for the calculating of electrical conductivity.

| | **Length (cm)** | **Width (cm)** | **Thickness (cm)** | **Cross-sectional area ($cm^2$)** | **Electrical conductance (S)** | **Electrical conductivity (S/cm)** |
|---|---|---|---|---|---|---|
| **Compound 1** | 0.286 | 0.100 | 0.0166 | 1.66e-3 | 7.14e-12 | 1.23e-9 |
| **Compound $I_3^-$@1** | 0.314 | 0.198 | 0.0288 | 5.70e-3 | 9.98e-10 | 5.50e-8 |
| **Compound 2** | 0.390 | 0.178 | 0.0106 | 1.89e-3 | 5.59e-12 | 1.16e-9 |
| **Compound $I_3^-$@2** | 0.250 | 0.136 | 0.0109 | 1.48e-3 | 9.93e-10 | 1.68e-7 |

$$\sigma = \mathrm{G}\frac{L}{A}$$

Electrical conductivity, σ, measures a material's ability to conduct electrical current. Measuring σ typically requires incorporating the material of interest into an electronic device, typically a resistor, and measuring the electrical conductance (G), length (L), and cross-sectional area (A) of the conduction channel.

**Table S8** Crystal date and structure refinement parameters for compound **2-open** and **2-close**.

| | **2-open** | **2-close** |
|---|---|---|
| Empirical formula | $C_{72}H_{48}O_{16}In_2N_2S_8$ | $C_{72}H_{48}O_{16}In_2N_2S_8$ |
| Formula weight | 1684.25 | 1683.33 |
| Temperature (K) | 80 | 273 |
| Crystal system | Triclinic | Triclinic |
| Wavelength (Å) | 1.54178 | 1.54178 |
| Space group | $P\bar{1}$ (No. 2) | $P\bar{1}$ (No. 2) |
| Unit cell dimensions (Å, °) | | |
| a | 12.1632(13) | 14.611(3) |
| b | 15.3236(18) | 14.811(3) |
| c | 30.622(2) | 30.363(8) |
| α | 83.6933(13) | 93.057(4) |
| β | 88.5300(19) | 93.057(4) |
| γ | 87.9124(18) | 117.9800 |
| Volume ($Å^3$) | 5667.8(10) | 5772(2) |
| Z | 2 | 2 |
| Calculated density ($gcm^{-3}$) | 0.986 | 0.969 |
| F(000) | 1696 | 1696 |
| Absorption coefficient, $\mu/mm^{-1}$ | 4.992 | 4.902 |
| No. of reflections measured | 65995 | 20772 |
| No. of independent reflections | 17923 | 10943 |
| θ (°) | 2.9-62.5 | 2.9-52.5 |
| $R_{int}$ | 0.034 | 0.073 |
| $R_1, wR_2\ [I \geq 2\sigma\ (I)]$ | 0.0411/ 0.1012 | 0.0900/ 0.2222 |
| $R_1, wR_2$ *[all data]* | 0.0579/ 0.1037 | 0.1488/ 0.2691 |
| GOF | 1.015 | 1.20 |
| Largest diff. peak and hole($eÅ^{-3}$) | 0.64 /-0.53 | 1.35 / -1.13 |

$^{a}R1 = \Sigma||F_o| - |F_c||/\Sigma|F_o|$, $wR_2 = [\Sigma w(F_o^2 - F_c^2)^2/\Sigma w(F_o^2)^2]^{1/2}$.

**Table S9** Crystal date and structure refinement parameters for compound **TEA@2** and **TBA@2**.

| | **TEA@2** | **TBA@2** |
|---|---|---|
| Empirical formula | $C_{88.43}H_{82.83}O_{18}In_2N_4S_8$ | $C_{90}H_{82}O_{18}In_2N_3S_8$ |
| Formula weight | 1981.87 | 1979.79 |
| Temperature (K) | 103 | 80 |
| Crystal system | Triclinic | Triclinic |
| Wavelength (Å) | 1.54178 | 1.54178 |
| Space group | $P\bar{1}$ (No. 2) | $P\bar{1}$ (No. 2) |
| Unit cell dimensions (Å, °) | | |
| a | 12.0244(6) | 12.1284(5) |
| b | 15.4176(8) | 15.2917(7) |
| c | 30.7305(14) | 30.6531(13) |
| α | 81.8987(18) | 83.195(2) |
| β | 88.5201(12) | 88.220(2) |
| γ | 88.1230(14) | 88.062(2) |
| Volume ($Å^3$) | 5635.9(5) | 5639.7(4) |
| Z | 2 | 2 |
| Calculated density ($gcm^{-3}$) | 1.164 | 1.166 |
| F(000) | 2023 | 2026 |
| Absorption coefficient, $\mu/mm^{-1}$ | 5.109 | 5.103 |
| No. of reflections measured | 61827 | 19925 |
| No. of independent reflections | 19451 | 19925 |
| θ (°) | 1.5-67.2 | 2.9-68.8 |
| $R_{int}$ | 0.043 | 0.017 |
| $R_1$, $wR_2$ $[I \geq 2\sigma (I)]$ | 0.0502 / 0.1200 | 0.0495 / 0.1269 |
| $R_1$, $wR_2$ *[all data]* | 0.0745 / 0.1256 | 0.0622 / 0.1309 |
| GOF | 1.04 | 0.93 |
| Largest diff. peak and hole($eÅ^{-3}$) | 0.63 /-0.67 | -1.45 / 1.84 |

[a] $R1 = \Sigma||F_o| - |F_c||/\Sigma|F_o|$, $wR_2 = [\Sigma w(F_o^2 - F_c^2)^2/\Sigma w(F_o^2)^2]^{1/2}$.

## Supplementary Methods

**Synthesis of $H_4$TTFTB.** $H_4$TTFTB (tetrathiafulvalene tetrabenzoate) was prepared adopting the procedure reported by Yorrimitsu and our group[1,2].

**Solid state cyclic voltammetry.** Solid state cyclic voltammetry measurements were performed in n-$Bu_4NPF_6/CH_3CN$ electrolyte using a BASi Epsilon electrochemical analyser and three electrode system. Argon was bubbled through solutions of 0.1 M $[(n\text{-}C_4H_9)_4N]PF_6$ dissolved in distilled $CH_3CN$. The CVs were recorded using a glassy carbon working electrode (1.5 mm diameter), a platinum wire auxiliary electrode and an $Ag/Ag^+$ wire quasi-reference electrode. The sample was mounted on the glassy carbon working electrode by dipping the electrode into a paste made of the powder sample in $CH_3CN$. Ferrocene was added as an internal standard upon completion of each experiment. All potentials are reported in mV versus $Fc/Fc^+$ couple.

**Solid-state UV-Vis-NIR spectra.** Solid-state UV-Vis-NIR spectra were obtained on the samples at room temperature using a CARY5000 Spectrophotometer equipped with a Harrick Praying Mantis accessory over the wavenumber range 5000-40000 $cm^{-1}$. $BaSO_4$ was used for the baseline. Spectra are reported as the Kubelka-Munk transform, where $F(R) = (1-R)^2/2R$ ($R$ is the diffuse reflectance of the sample as compared to $BaSO_4$). Solution-state UV-vis spectra were measured on a UV-3100 spectrophotometer.

**Solid-state diffuse reflectance Vis-NIR spectroelectrochemistry.** Solid-state diffuse reflectance Vis-NIR spectroelectrochemistry was obtained in situ in a 0.1 M $[(n\text{-}C_4H_9)_4N]PF_6/CH_3CN$ electrolyte over the range 5000-25000 $cm^{-1}$ using a Harrick Omni Diff Probe attachment and a custom built solid-state spectroelectrochemical cell[3]. The cell consisted of a Pt wire counter electrode and a $Ag/Ag^+$ quasi reference electrode. The solid sample was immobilized onto a 0.1 mm thick Indium-Tin-Oxide (ITO) coated glass slide (which acted as the working electrode) using a thin strip of Teflon tape. The applied potential was controlled using an eDAQ potentiostat.

**Conductivity test.** The conductivity of the samples was obtained from Keithley 2400 source meter on CRX-4K High Performance Closed Cycle Refrigerator-based Probe Station at room temperature. The sample powders were cold-pressed under pressure of 10 Mpa into pellets, which were connected by the conductive carbon adhesive with the “two probe method”. The conductivity σ could be expressed as, $\sigma = G \cdot L / A$, where L and G are the thickness, electrical conductance of the pellet, respectively, and A is the area of the conductive carbon adhesive. All of the current-voltage (I-V) measurements were performed in ambient conditions by sweeping the voltage from -1.5 V to 1.5 V.

**X-ray Structure Studies.** Single-crystal X-ray crystallographic studies: Data of **1**, $I_3^-$@**1**, **2** and $I_3^-$@**2** were collected on a Bruker D8 Venture diffractometer outfitted with a PHOTON-100 CMOS detector using monochromatic microfocus Mo-$K_\alpha$ radiation (λ= 0.71073 Å) that was operated at 50 kV and 40 mA at 153 K by chilled nitrogen flow controlled by a KRYOFLEX II low temperature attachment. Data of **2-close**, **2-open**, TEA@**2** and TBA@**2** were collected on a Bruker D8 Venture diffractometer using monochromatic microfocus Cu-$K_\alpha$ radiation (λ= 1.54184 Å). Raw data collection and reduction were done using APEX3 software[4]. Adsorption corrections were applied using the SADABS routine. The structures were solved by direct methods and refined by full-matrix least-squares on $F^2$ using the SHELXTL software package[5]. Non-hydrogen atoms were refined with anisotropic displacement parameters during the final cycles. Hydrogen atoms of $H_4$TTFTB were calculated in

ideal positions with isotropic displacement parameters; those of dimethylammonium for compounds **1**, $I_3^-$@**1**, **2** and $I_3^-$@**2** were originally found from electron density peaks and then refined with restrictions. Free solvent molecules of dimethylformamide or water for compounds **1**, $I_3^-$@**1**, **2** and $I_3^-$@**2** were highly disordered, and were unsuccessful to locate and refine the solvent peaks. The diffuse electron densities resulting from these residual solvent molecules were removed from the data set using the SQUEEZE routine of PLATON and refined further using the data generated.[6] The contents of the solvent region are not represented in the unit cell contents in crystal data. Attempts to determine the final formula of such compounds from the SQUEEZE results combined with elemental analysis and TGA data were not successful because the volatility of the crystallization solvents during measurements prevented accurate data from being obtained. So, the final formula of compounds **1**, $I_3^-$@**1**, **2** and $I_3^-$@**2** were calculated from the elemental analysis, TGA data.

## Supplementary References


1 Chen, B. *et al.* Crystal Structures, Gas Adsorption, and Electrochemical Properties of Electroactive Coordination Polymers Based on the Tetrathiafulvalene-Tetrabenzoate Ligand. *Cryst. Growth & Des.* **15**, 1861-1870, doi:10.1021/acs.cgd.5b00014 (2015).

2 Mitamura, Y., Yorimitsu, H., Oshima, K. & Osuka, A. Straightforward access to aryl-substituted tetrathiafulvalenes by palladium-catalysed direct C–H arylation and their photophysical and electrochemical properties. *Chem. Sci.* **2**, 2017, doi:10.1039/c1sc00372k (2011).

3 Usov, P. M., Fabian, C. & D'Alessandro, D. M. Rapid determination of the optical and redox properties of a metal-organic framework via in situ solid state spectroelectrochemistry. *Chem. Comm.* **48**, 3945-3947, doi:10.1039/c2cc30568b (2012).

4 Görbitz, C. H. What is the best crystal size for collection of X-ray data? Refinement of the structure of glycyl-L-serine based on data from a very large crystal. *Acta Crystallogr. B* **55**, 1090-1098, doi:10.1107/s0108768199008721 (1999).

5 Sheldrick, G. M. A short history of SHELX. *Acta crystallogr. A* **64**, 112-122, doi:10.1107/S0108767307043930 (2008).

6 Spek, A. L. Single-crystal structure validation with the programPLATON. *J. Appl. Crystallogr.* **36**, 7-13, doi:10.1107/s0021889802022112 (2003).
